# Author Correction: Revisiting the soil carbon saturation concept to inform a risk index in European agricultural soils

**DOI:** 10.1038/s41467-026-68444-x

**Published:** 2026-01-14

**Authors:** T. S. Breure, D. De Rosa, P. Panagos, M. F. Cotrufo, A. Jones, E. Lugato

**Affiliations:** 1 European Commission, Joint Research Centre, Ispra, Italy; 2 Department of Agriculture, Forestry, Food and Environmental Sciences, University of Basilicata, Potenza, Italy; 3 Department of Soil and Crop Science and Natural Resource Ecology Laboratory, Colorado State University, Fort Collins, USA

**Keywords:** Carbon cycle, Environmental impact, Carbon cycle

Correction to: *Nature Communications* 10.1038/s41467-025-57355-y, published online 18 March 2025

In the version of this article initially published, there was an error in the fifth paragraph of the “Estimating the effective MAOC capacity by clusters” section, where in the text now reading “We first converted all parameter estimates to the same unit (g MAOC kg^−1^ fine fraction),” the text “kg^−1^ fine fraction” originally read “in kg^−1^ soil.” Further in the same paragraph, the text now reading “The mean estimate for the NBL method was 28.5 ± 6 (SD) and spanned the smallest range across pedo-climatic zones (Supplementary Figs. 5–7). The upper limit parameter values for the NBL and PBL methods (41, 45, respectively) were lower than for BL (62), which is lower than previous estimates for 2:1 mineral dominated soils” replaces the original “The mean estimate for the NBL method was 40 ± 17 (SD) and spanned the largest range across pedo-climatic zones (Supplementary Figs. 5–7). The upper limit parameter values for the BL and PBL methods (62, 45, respectively) were lower than for NBL (79), which approximated previous estimates for 2:1 mineral dominated soils.” In the sixth paragraph of the same section, in the sentence now reading “For example, recent studies^42^ pointed to soils with higher MAOC content that may be formed under particular conditions (e.g., very high clay, hydromorphic conditions) and can exceed 50 g MAOC kg^-1^ soil…” now replaces the original “For example, recent studies^42^ pointed to soils with higher MAOC content that may be formed under particular conditions (e.g., very high clay, hydromorphic conditions) and can exceed 50 g kg^-1^ MAOC^5^.” Also, a new sentence has been added at the end of that paragraph: “The NBL method notably led to lower estimates (29 g MAOC kg^-1^ fine fraction)”. Captions to Supplementary Figs. 5–7, 14 have been updated similarly. The changes have been made in the HTML and PDF versions of the article.

