# Supplementary material for: Revisiting the soil carbon saturation concept to inform a risk index in European agricultural soils
Source: Nat Commun. 2025 Mar 18;16:2538. doi: 10.1038/s41467-025-57355-y (PMC11920252; doi:10.1038/s41467-025-57355-y)
Supplement: Supplementary file 1 — Supplementary Information [file 41467_2025_57355_MOESM1_ESM.pdf]

Supplementary material

# Revisiting the soil carbon saturation concept to inform a risk index in European agricultural soils

Breure, T.S.[1], De Rosa, D.[2], Panagos, P.[1], Cotrufo, M.F.[3], Jones, A.[1] and Lugato, E.[1]

[1]European Commission, Joint Research Centre, Ispra, Italy

[2]School of Agriculture, Forestry, Food and Environmental Sciences, University of Basilicata, Potenza, Italy

[3] Department of Soil and Crop Science and Natural Resource Ecology Laboratory, Colorado State University, Fort Collins, United States

## Schematic flowchart

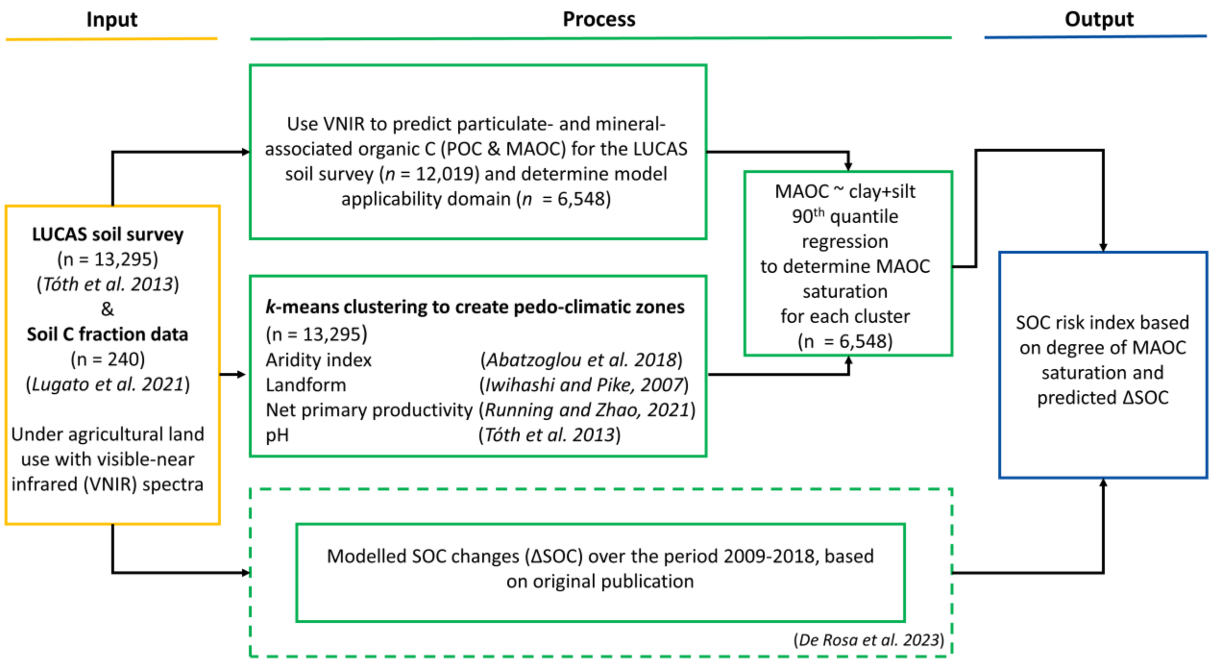


**Supplementary Figure 1 | Flow chart of the key research components** regarding the dataset input, the process of clustering, soil carbon (C) fraction estimation, calculation of mineral-associated organic carbon (MAOC) saturation, estimation of the soil organic carbon (SOC) changes, and finally the output as a SOC risk index. Sample sizes for the different steps are given by (n) and are also listed in the methodology section of the main text[1–6].

## Calibration regression and model applicability domain

We performed an exploratory analysis in order determine which calibration regression method to use. We followed the standard procedure by using the Kennard-Stone sampling method on the Euclidean distance of the principal components from the 1st derivative VNIR spectra that explained 99% of the variance (6 principal components) to split the dataset into 75% of the samples for calibration and 25% for validation[7]. We considered three different regression methods. Partial least squares regression (PLS), which is considered a reference method in the chemometrics literature. A rules-based regression, Cubist, which has shown to provide interpretable results and good prediction performance in soil spectroscopy[8]. Local partial least squares regression, where the nearest neighbors of a sample to be predicted are selected based on spectral similarities and PLS functions are built based on this subset[9]. The decision on with which method to proceed was made based on the mean prediction metrics for the validation set. These metrics where: the root mean squared error (RMSE), bias, Lin's concordance correlation coefficient (CCC)[10] and the ratio of the standard prediction error over the inter-quartile range (RPIQ)[11].

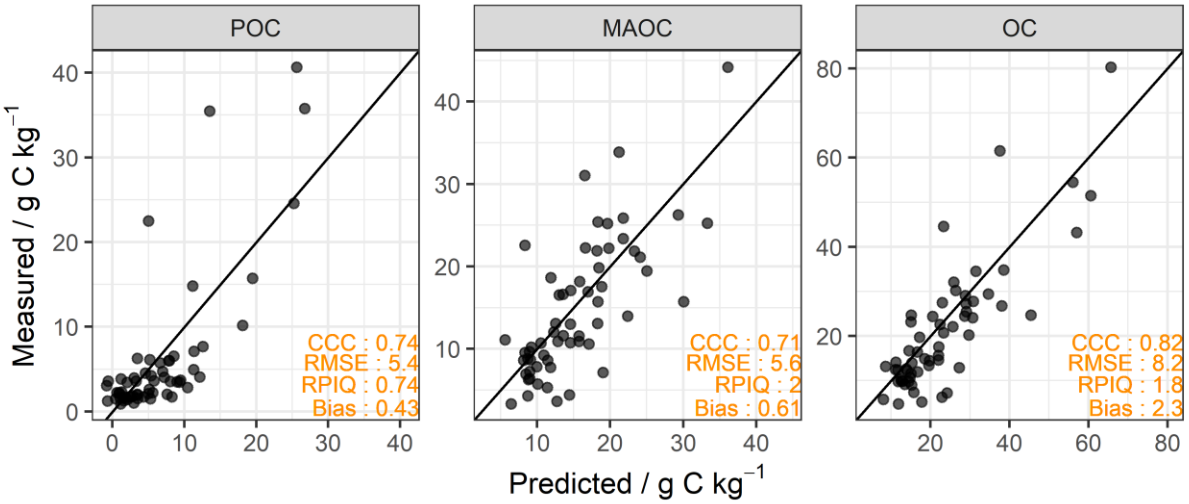


**Supplementary Figure 2 | Predicted versus measured particulate organic carbon (POC), mineral-associated carbon (MAOC) and soil organic carbon (SOC) for the validation set**. Results are shown from the local partial least squares regression method that was used in the main text.

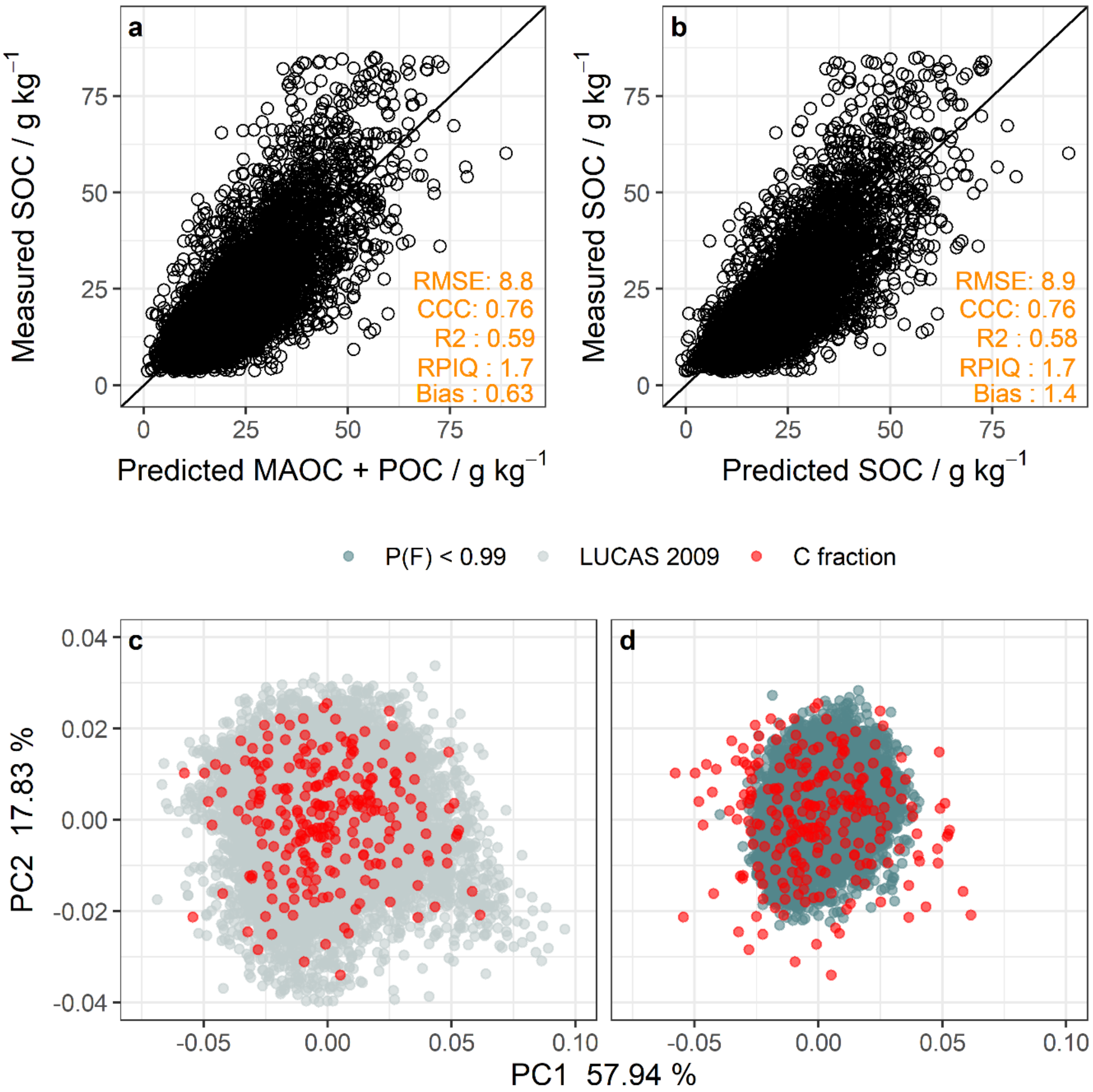


**Supplementary Figure 3 | Prediction validation metrics and the model applicability domain** Top row figures (**a** and **b**): predicted versus measured soil organic carbon (SOC) for the LUCAS 2009 where the F-ratio probability < 0.99 for both particulate- and mineral-associated organic carbon (POC and MAOC) predictions (P(F) < 0.99)[12–14] (n = 6,548). **a**. predicted SOC as the sum of MAOC + POC, and **b**. for predicted bulk SOC. Bottom row figures (**c** and **d**): the joint distribution of the first two principle components from the soil spectra for **c**. LUCAS 2009 under agricultural land use (n = 12,019), and the soil C fraction calibration data (n = 240) and **d**. LUCAS 2009 subset to P(F) < 0.99 (n = 6,548). Where the prediction metrics are listed as metrics as follow: the root mean squared error (RMSE), bias, Lin's concordance correlation coefficient (CCC)[10] the coefficient of determination (R2) and the ratio of the standard prediction error over the inter-quartile range (RPIQ)[11].

## MAOC and POC in relation to total SOC changes

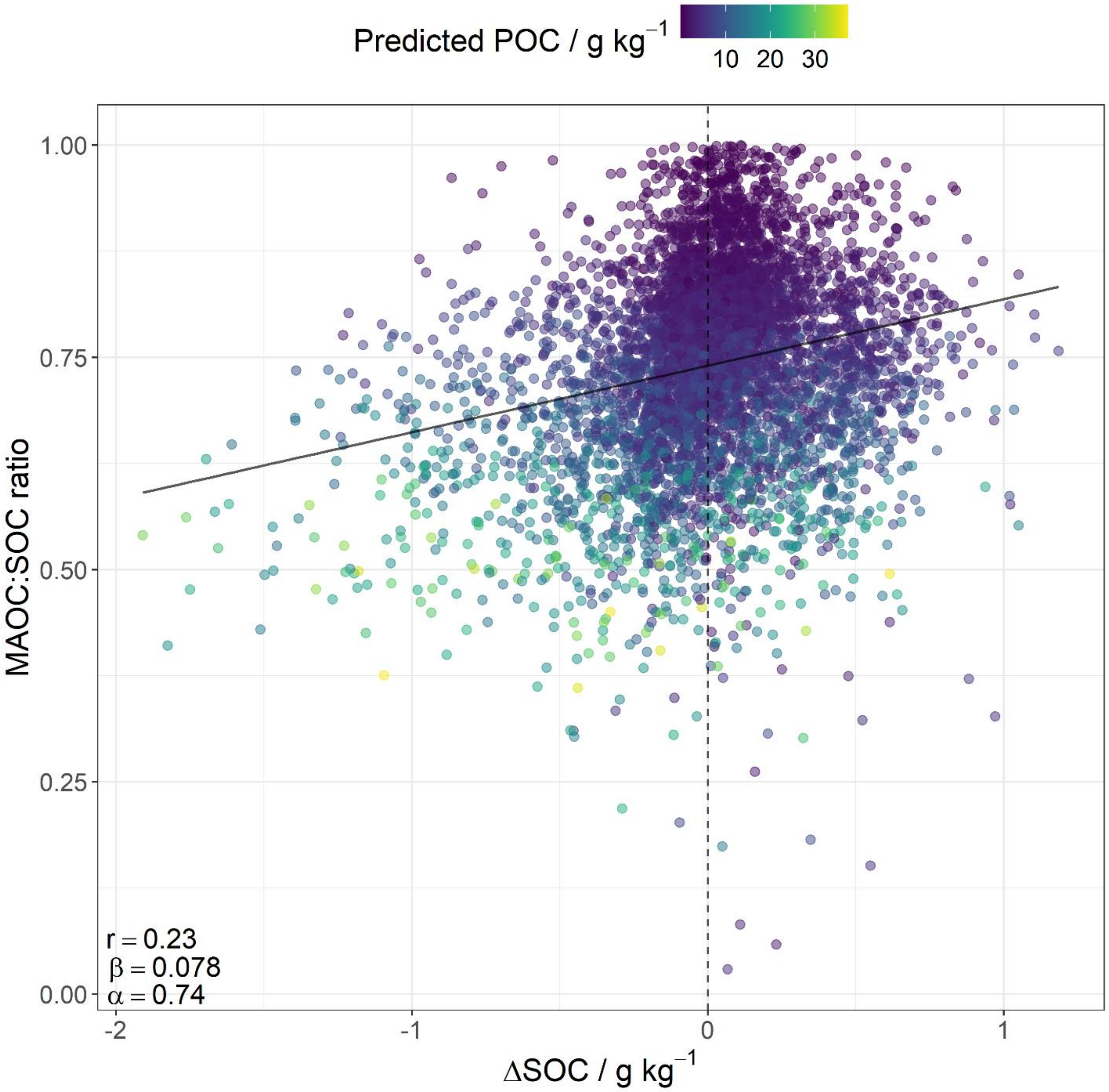


**Supplementary Figure 4 | Predicted changes in soil organic carbon (ΔSOC) versus the predicted mineral associated organic carbon fraction of total soil organic carbon [MAOC:SOC ratio (with SOC=MAOC + POC)]** colored by the soil particulate organic carbon (POC) concentration. *r* is Pearson's correlation coefficient, α and β are the intercept and slope estimates of the linear least-squares model (solid black line). n = 5,482.

**Supplementary Table 1 | Summary table for linear model slope terms (β) accompanying Fig. 2 in the main text** Cluster ID is the pedo-climatic cluster association, SE the standard error, Df the degrees of freedom, lower- and upper ranges of the 95% confidence interval (CI 95%). n = 5,482.

| Cluster ID | B | SE | Df | Lower.CI 95% | Upper.CI 95% |
|---|---|---|---|---|---|
| Cluster 1 | 0.09 | 0.03 | 5450 | 0.03 | 0.14 |
| Cluster 2 | 0.15 | 0.02 | 5450 | 0.11 | 0.19 |
| Cluster 3 | 0.08 | 0.02 | 5450 | 0.05 | 0.12 |
| Cluster 4 | 0.11 | 0.03 | 5450 | 0.05 | 0.17 |
| Cluster 5 | 0.10 | 0.02 | 5450 | 0.05 | 0.15 |
| Cluster 6 | 0.09 | 0.02 | 5450 | 0.05 | 0.12 |
| Cluster 7 | 0.06 | 0.02 | 5450 | 0.03 | 0.10 |
| Cluster 8 | 0.08 | 0.01 | 5450 | 0.06 | 0.11 |
| Cluster 9 | 0.08 | 0.02 | 5450 | 0.05 | 0.11 |
| Cluster 10 | 0.09 | 0.01 | 5450 | 0.07 | 0.12 |
| Cluster 11 | 0.08 | 0.02 | 5450 | 0.05 | 0.12 |
| Cluster 12 | 0.15 | 0.02 | 5450 | 0.11 | 0.18 |
| Cluster 13 | 0.11 | 0.02 | 5450 | 0.06 | 0.16 |
| Cluster 14 | 0.04 | 0.02 | 5450 | 0.01 | 0.08 |
| Cluster 15 | 0.06 | 0.03 | 5450 | 0.01 | 0.11 |
| Cluster 16 | 0.05 | 0.01 | 5450 | 0.02 | 0.08 |

**Supplementary Table 2 | ANOVA table for pedo-climatic cluster as an interaction term with ΔSOC in the MAOC:SOC~ΔSOC linear model in Fig. 2**

| Term | Df | Sumsq | Meansq | Statistic | *p*-value |
|---|---|---|---|---|---|
| ΔSOC | 1 | 3.93 | 3.93 | 333.06 | 2.84E-72 |
| Cluster | 15 | 7.75 | 0.52 | 43.82 | 1.05E-122 |
| ΔSOC *x* Cluster | 15 | 0.49 | 0.03 | 2.77 | 0.000267 |
| Residuals | 5450 | 64.28 | 0.01 | - | - |

# Calculating the effective mineral-associated organic carbon capacity

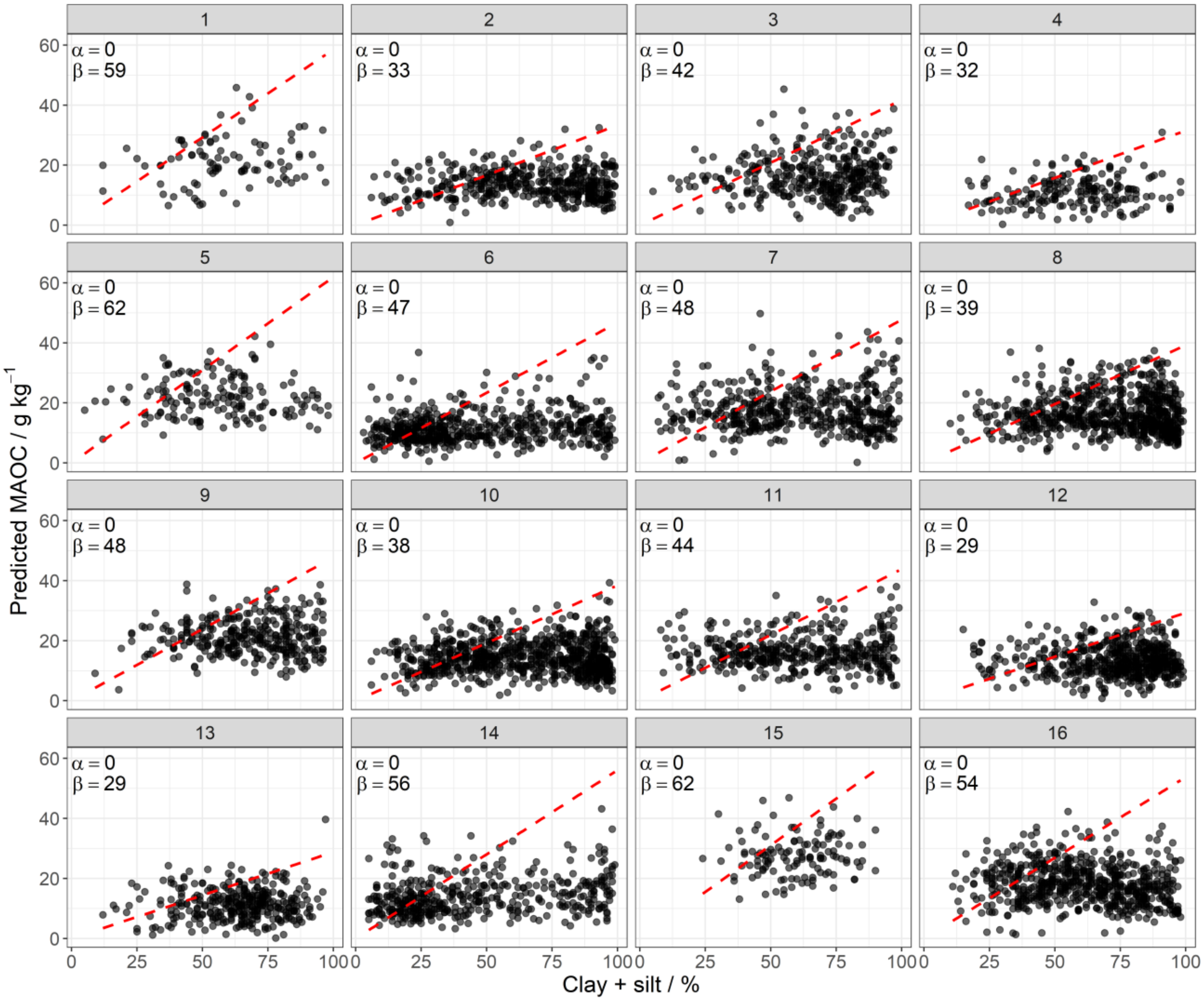


**Supplementary Figure 5 | Linear 90th quantile regression to determine the effective mineral-associated organic carbon (MAOC) capacity** for the boundary line method (BL) by each pedo-climatic cluster, the intercept was restricted to 0. For ease of cross-comparison with alternative methods, parameter estimates are reported in g MAOC kg$^{-1}$ fine fraction. (n = 6,548)

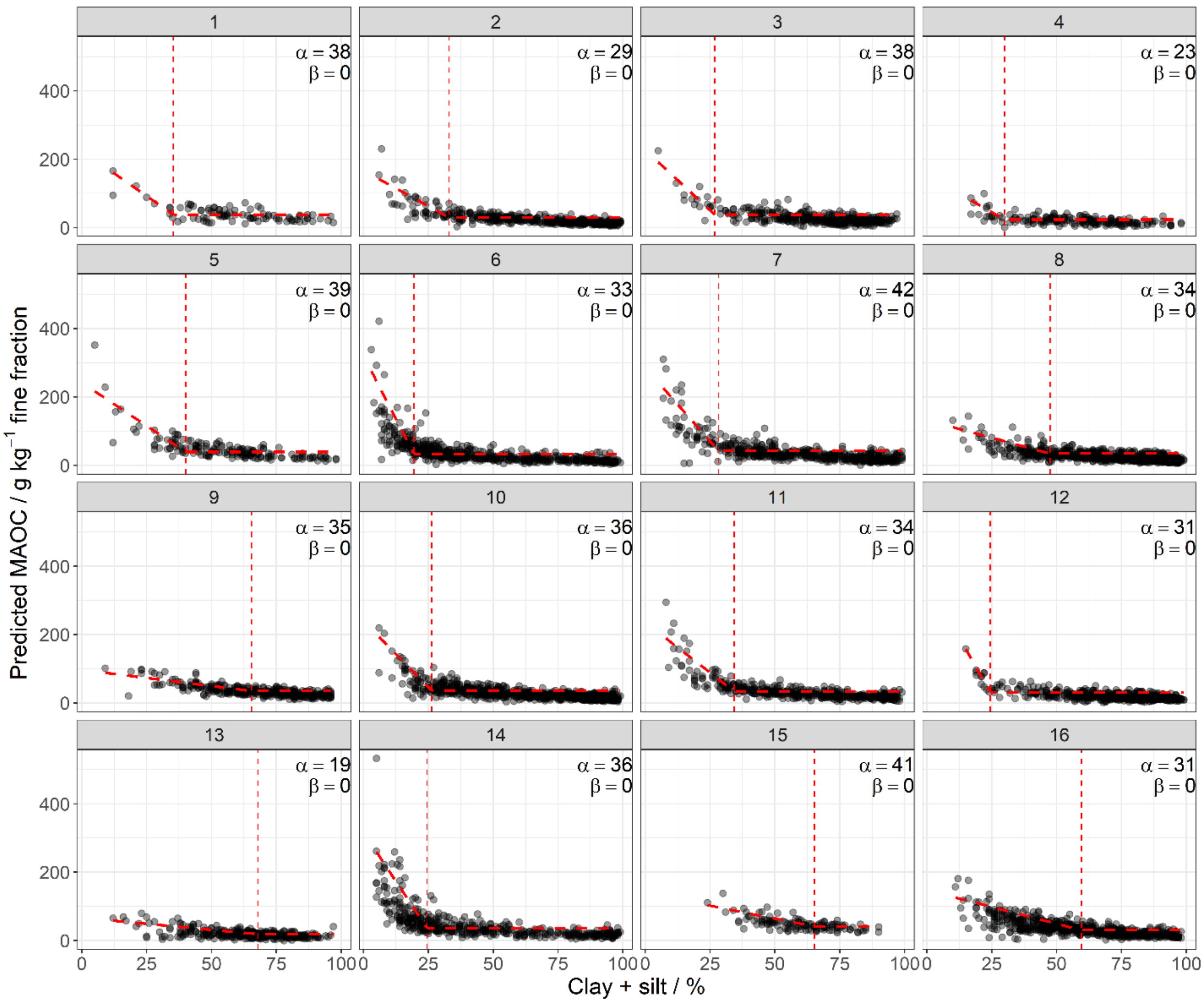


**Supplementary Figure 6 | Piece-wise linear 90$^{th}$ quantile regression to determine the effective mineral-associated organic carbon (MAOC) capacity** for the piece-wise boundary line method (PBL), slope of the second linear equation (after the breakpoint, indicated by the vertical dashed line) was restricted to 0. For ease of cross-comparison with alternative methods, parameter estimates are reported in g MAOC kg$^{-1}$ fine fraction. (n = 6,548)

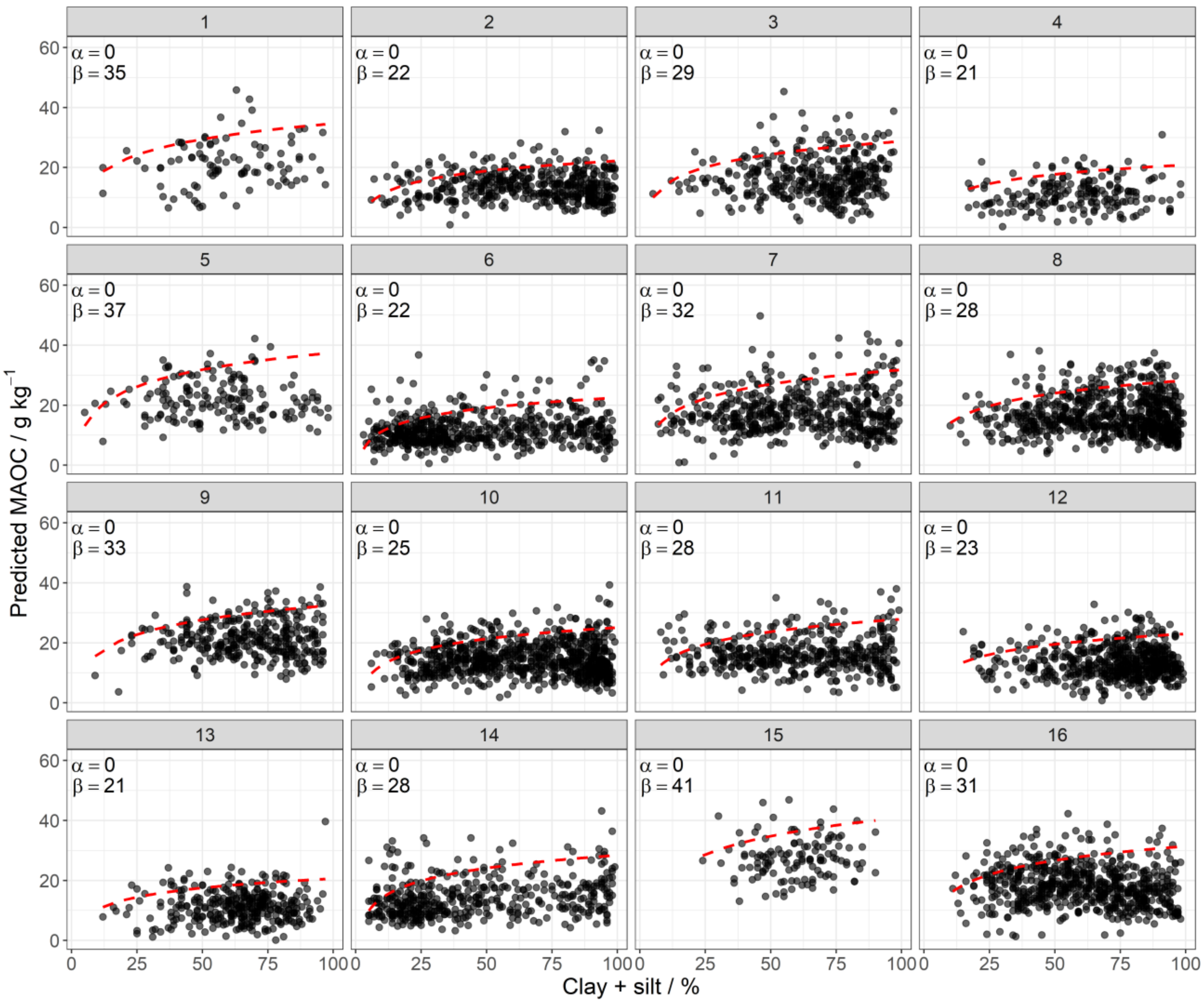


**Supplementary Figure 7 | Non-linear 90$^{th}$ quantile regression to determine the effective mineral-associated organic carbon (MAOC) capacity** for the non-linear boundary line method (NBL), intercept was restricted to 0. For ease of cross-comparison with alternative methods, parameter estimates are reported in g MAOC $kg^{-1}$ fine fraction. Given that the NBL method assumes a changing MAOC content across the fine-fraction content, the β parameter can be considered the value at 100% clay + silt content. (n = 6,548)

## Sensitivity analysis for *k*-means clustering procedure

In order to assess the effect of clustering variables (aridity, net primary productivity, pH in $H_2O$, landform) on the estimated effective mineral-associated organic carbon (MAOC) capacity, we have done a sensitivity analysis for the *k*-means clustering procedure as implemented in the manuscript. Specifically, we have iteratively removed each of the four covariates used in the clustering, estimated the effective MAOC capacity and assessed the distribution of the effective MAOC capacity across clusters. For the purpose of the sensitivity analysis, we have estimated the effective MAOC capacity by the NBL method only, given that this method gave the best fit for our data.

Our hypothesis was that the spread in β parameters, indicating the effective MAOC capacity, should be reduced when excluding a cluster covariate that helps to distinguish between the theoretical (based on fine fraction mineralogy only) and the effective MAOC capacity (considered as an ecosystem property, accounted for by controlling factors). That is, if the cluster covariates have an effect on controlling effective MAOC capacity, their exclusion would narrow the distribution in β parameters. In case they do not have an effect, the distribution across the clusters would be similar in the leave-one-covariate-out experiment compared to the original.

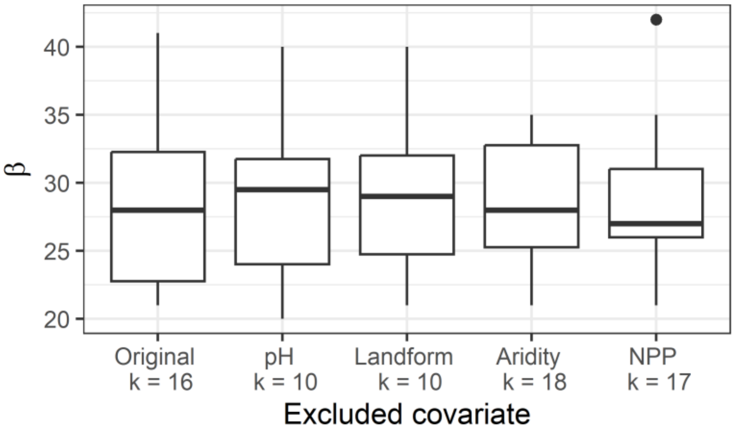


**Supplementary Figure 8 | Distribution of β parameters across all clusters from the leave-one-covariate-out sensitivity analysis.** 'Original' is as reported in the main text. The other boxplots show the distribution when that covariate has been excluded from the clustering, where *k* indicates the total number of clusters. Model fitting was done as per the NBL method, specified in Figure 3 in the main text. The center line of the boxplots is the median, the boxplot lower- and upper limits are equal to the first and third quartiles, respectively. The upper whisker extends 1.5 times the inter-quartile range from the upper limit, vice versa for the lower whisker.

Our findings showed that this was not the case (Supplementary Fig. 8): the inter-quartile range of the β parameter distribution across all clusters was reduced when removing factors from the k-means clustering. This indicates that these covariates help to distinguish between the theoretical (based on fine fraction only) and the effective MAOC capacity.

There are two main effects of covariates on reducing the theoretical maximum to effective maximum MAOC capacity: i.) aridity and NPP have a larger effect on the β parameter distribution, ii.) pH and landform also showed an effect although it was smaller. Whereas pH and landform tend to reduce the median effective MAOC capacity (i.e. their exclusion increases the median), aridity and NPP increase the median effective MAOC capacity.

## Comparison of results with Viscarra-Rossel et al. (2023) methodology

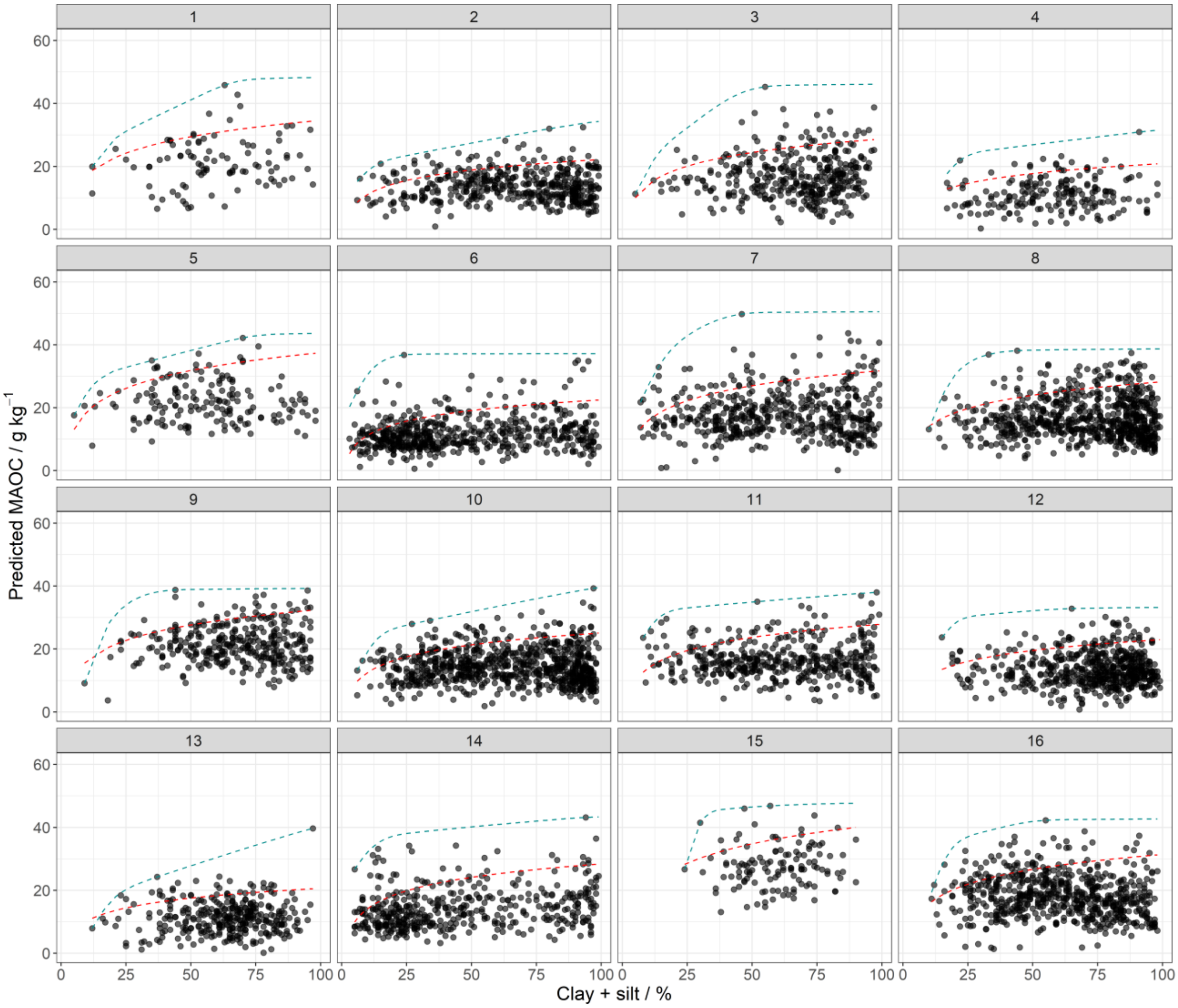


**Supplementary Figure 9 | Comparison of two methods to estimate the effective mineral-associated organic carbon (MAOC) capacity under the assumption of a texture-dependent MAOC concentration.** The red line corresponds to the non-linear 90$^{th}$ quantile regression to determine the effective MAOC capacity for the whole saturation method (NBL, Figure 3 in main text). The dashed blue line represents similar model as implemented in Viscarra-Rossel et al. (2023) to estimate the upper-boundary using frontier-line analysis. The method finds a locally weighted average based on fitting a bounded monotonic, and concave smoothing kernel ([15] for further details).

Given that the NBL method fits the 90$^{th}$ quantile[16,17], the estimated maximum MAOC capacity are substantially lower for some clusters compared to the frontier-line analysis. The frontier-line analysis fits exactly on the samples with high MAOC concentration, therefore providing an upper estimate for our dataset.

## Comparison with mineral-associated organic carbon data from the literature

We have also investigated the effect of including legacy carbon fraction data[18,19] on the parameter estimates (Supplementary Figs. 5—7).

We selected all points from Georgiou et al. (2022) according to two criteria: 1.) located within the EU but not part of LUCAS, 2.) were under cropland or grassland land use (n = 146)[19].

The Begill et al. (2023) data are available with protected coordinates (i.e. coordinates have been 'randomly' samples within a radius of 4 km from the sampling location), (n = 113)[18].

Figure 1 shows the boxplots of the MAOC from Georgiou et al. (2022) and Begill et al. (2023) compared to the data used in the manuscript (LUCAS VNIR, n = 6,548).

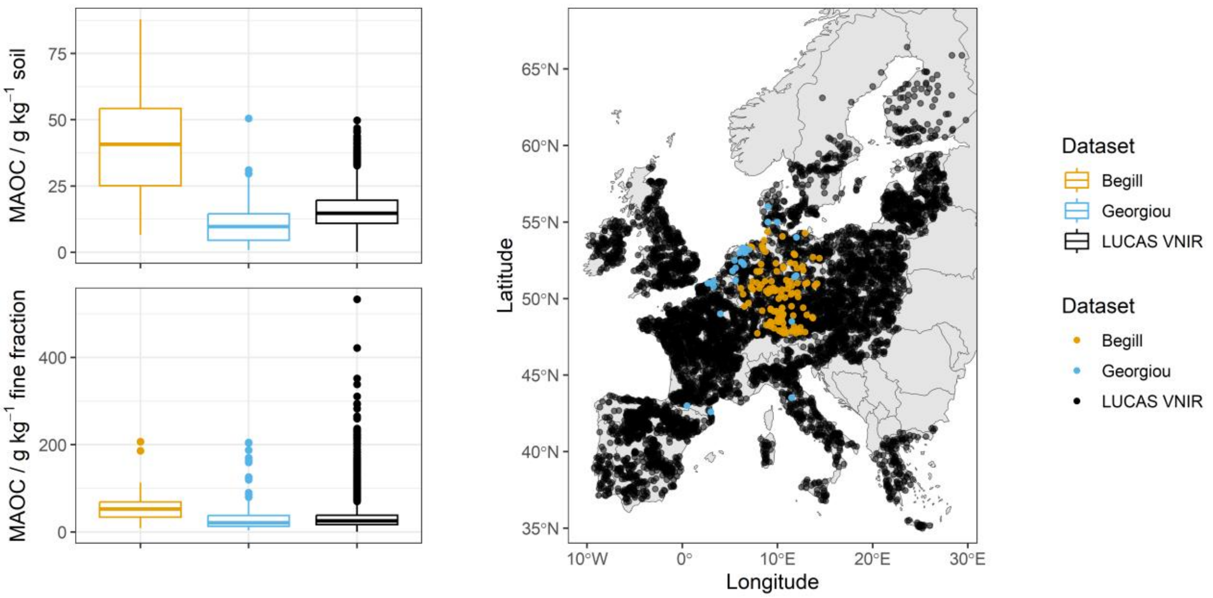


**Supplementary Figure 10 | Comparison of mineral-associated organic carbon (MAOC) data** based on LUCAS VNIR data used in our study (n = 6,548) and the datasets reported in Georgiou et al. (2022) (n = 146) and Begill et al. (2023) (n = 113). Vector map data used from the 'rnaturalearth' R package[20]. Copyright (CC0) (2025), (CRAN).

We extracted the pedo-climatic cluster association of these points based on the raster in Supplementary Figure 17 and then investigated the effect of including the legacy data by reproducing Supplementary Figures 5-7. Here we list a reproduction of Supplementary Figures 5-7 of the main text with the legacy data included.

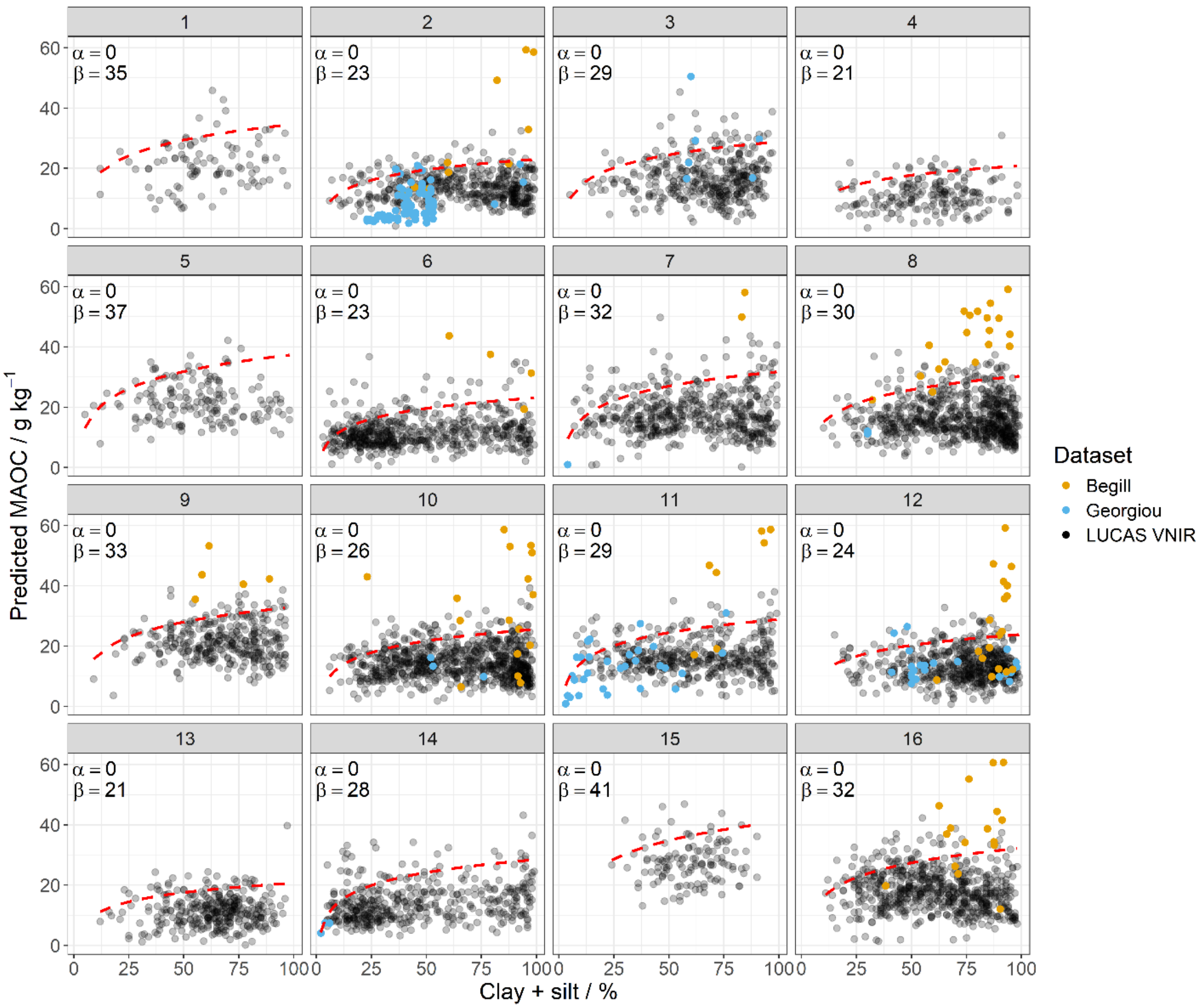


**Supplementary Figure 11 | Reproduction of Supplementary Figure 7: non-linear 90$^{th}$ quantile regression to determine the effective mineral-associated organic carbon (MAOC) capacity** for the non-linear boundary line method (NBL), the intercept was restricted to 0. Datasets correspond to Georgiou et al. (2022, n = 146), Begill et al. (2023, n = 113), and the LUCAS MAOC data based on visible near-infrared spectra (n = 6,548).

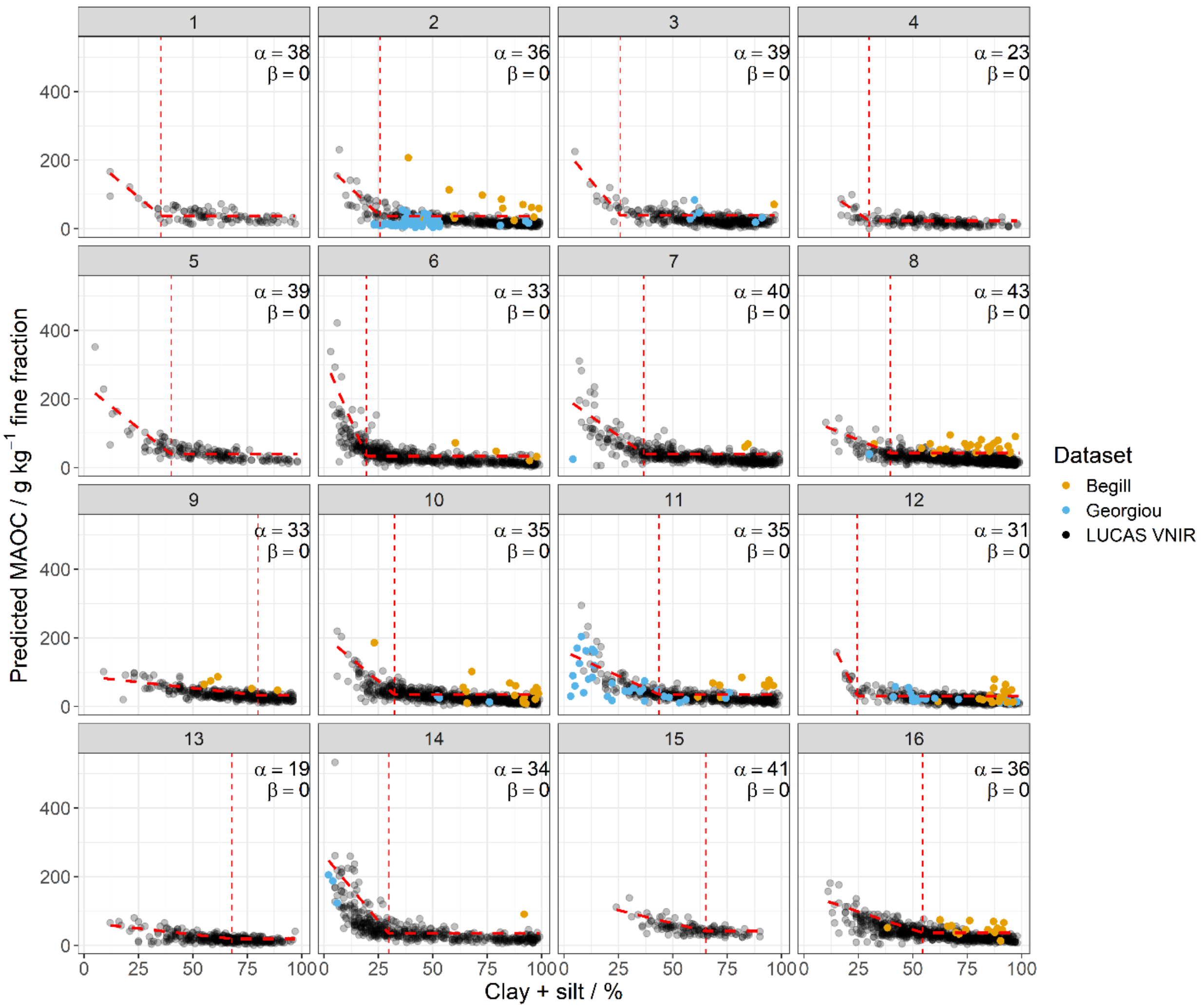


**Supplementary Figure 12 | Reproduction of Supplementary Figure 6: piece-wise linear 90th quantile regression to determine the effective mineral-associated organic carbon (MAOC) capacity** for the piece-wise boundary line method (PBL), slope of the second linear equation (after the breakpoint, indicated by the vertical dashed line) was restricted to 0. For ease of cross-comparison with other methods, the parameter estimates have been converted to g MAOC in kg$^{-1}$ of soil. Datasets correspond to Georgiou et al. (2022, n = 146), Begill et al. (2023, n = 113), and the LUCAS MAOC data based on visible near-infrared spectra (n = 6,548).

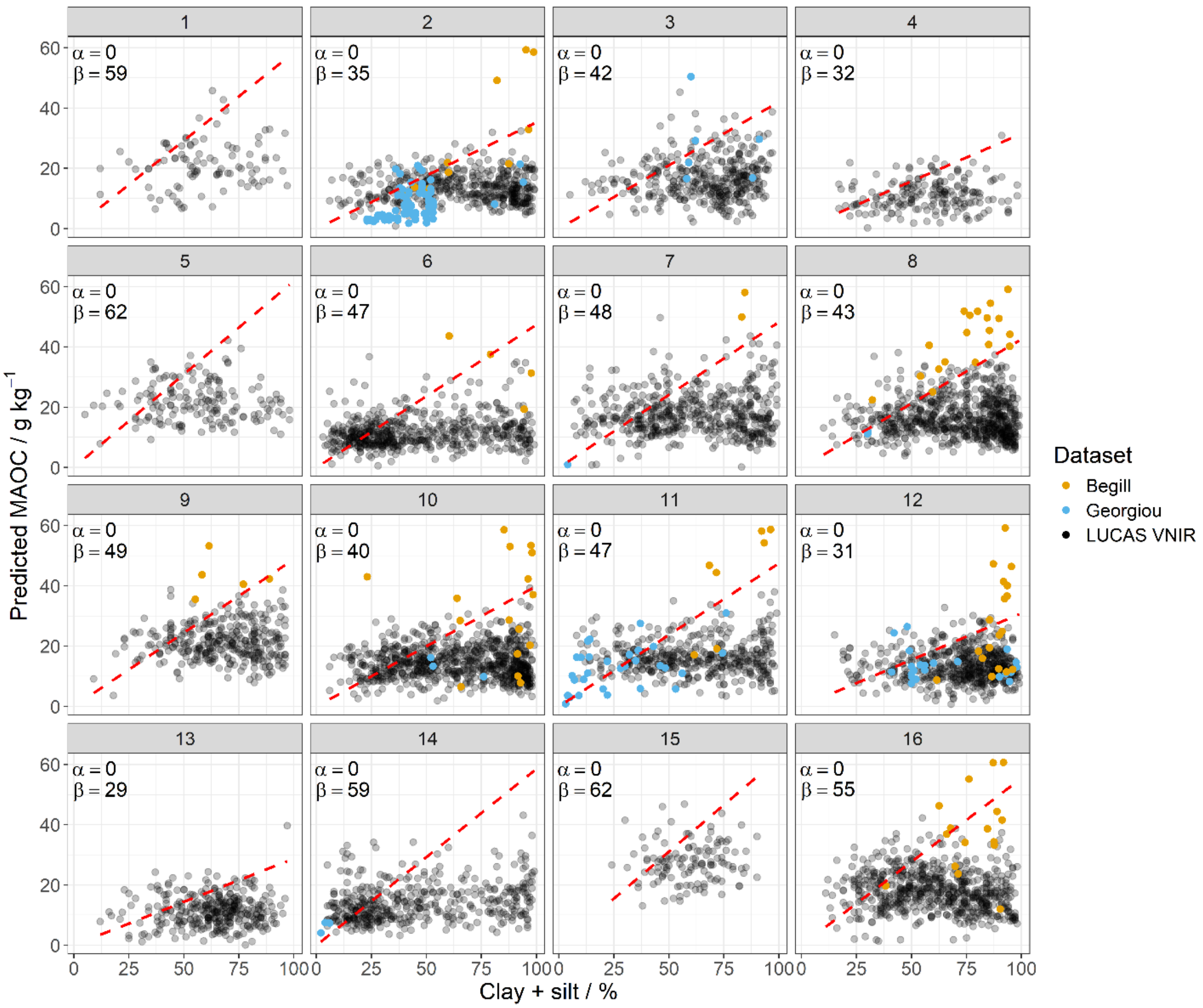


**Supplementary Figure 13 | Reproduction of Supplementary Figure 5: linear 90$^{th}$ quantile regression to determine the effective mineral-associated organic carbon (MAOC) capacity** for the boundary line method (BL), the intercept was restricted to 0. Datasets correspond to Georgiou et al. (2022, n = 146), Begill et al. (2023, n = 113), and the LUCAS MAOC data based on visible near-infrared spectra (n = 6,548).

Our short interpretation is as follows:

- The data from Georgiou et al. (2022) lay within the limits of the LUCAS VNIR data (Supplementary Fig. 10 and 11-13). The Begill et al. (2023) data lay partially within limits of the LUCAS VNIR data, particulary when MAOC was expressed as MAOC content in the fine fraction (lower left panel, Supplementary Figure 10).
- The inclusion of the legacy data affects parameter estimates for a minimal set of clusters only (mostly cluster 8 and 11), illustrating that separating the data by pedo-climatic clusters minimizes the leverage compared to pooling all the

data. That is, a large number of clusters remained unaffected by including the legacy data. For example cluster 4, located in the Iberian peninsula.

In the end, we decided not to include these legacy data in our analysis, due to the different sampling years, sampling depths and analytical methods used in the data of Georgiou et al. (2022) and Begill et al. (2023).

## Calculating the maximum mineral-associated organic carbon (MAOC) capacity

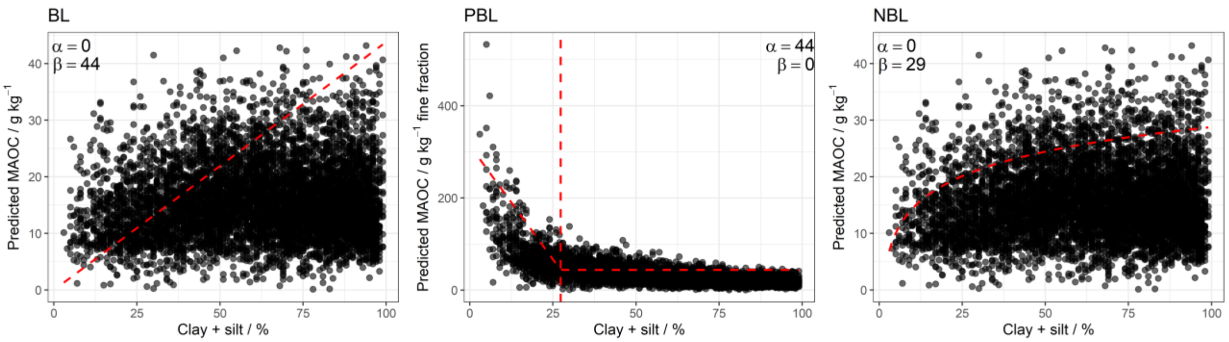


**Supplementary Figure 14 | Maximum mineral-associated organic carbon (MAOC) capacity estimation by regression on the fine fraction content, i.e. without accounting for pedo-climatic cluster association**. Panels represent the following methods: the boundary line (BL), piece-wise boundary line (PBL) and non-linear boundary line (NBL). See Figure 3 in the main text for details. All regressions were done for the 90th quantile with a forced intercept to 0 as per Feng et al. (2013). For ease of cross-comparison, parameter estimates are reported in g MAOC $kg^{-1}$ fine fraction. (n = 6,548)

# Degree of MAOC saturation before binning by fine fraction intervals

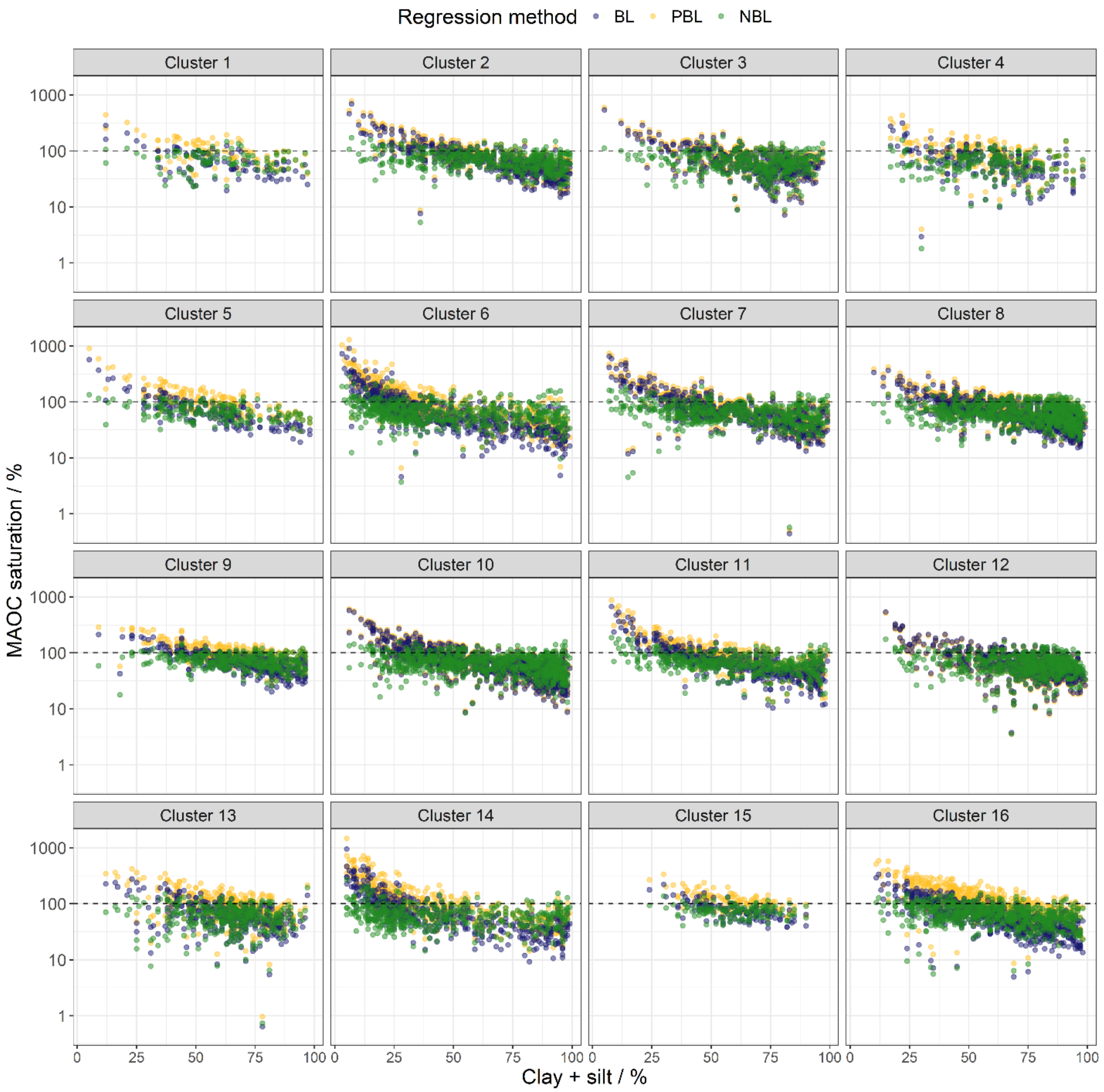


**Supplementary Figure 15 | Degree of mineral-associated organic carbon (MAOC) saturation (MAOC / effective MAOC capacity x 100%) as a function of fine fraction (clay + silt, %) for each cluster.** where the y-axis is on $\log_{10}$ scale. Values below 100% indicate a saturation deficit relative to the cluster-dependent effective MAOC capacity. Colors indicate three different regression methods to calculate maximum MAOC capacity. For details on the regression methods, see Figure 3 in the main text. n = 6,548.

## K-means clustering and upscaling

Since we wanted to calculate the SOC risk index for the entire ΔSOC raster published in De Rosa et al. (2024)[6], we used a random forest regression to predict the cluster association for each raster cell based on the aridity, pH, NPP and landform rasters. We computed the random forest regression between the cluster associations and the variables used in the *k*-means method (aridity, pH, NPP and landform), available at a gridded level across Europe. Estimates showed good correspondence based on the confusion matrix (Supplementary Fig. 16) and their distributions aligned relatively well with those of the LUCAS 2009 data which was used to formulate cluster associations (Supplementary Fig. 17).

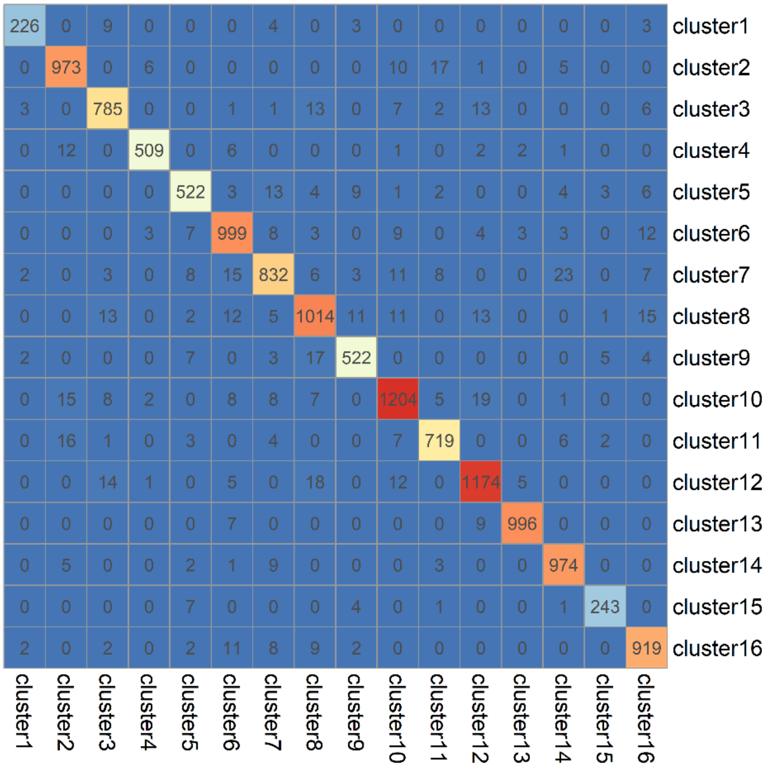


**Supplementary Figure 16 | Confusion matrix of the LUCAS 2009 dataset cluster associations based on the random forest regression.** X-axis are predicted cluster associations, y-axis are the cluster associations based on k-means. Numbers within cells represent the number of data points. n = 13,295.

For the clustering, we ran the *k*-means 100 times for different seeds (random number generators): within each iteration the *k*-means was ran 100 times for different random allocations of initial centers. Based on the results from the nested iteration and our selection criterion, 16 clusters was selected 95% of the time and 17 clusters 5%.

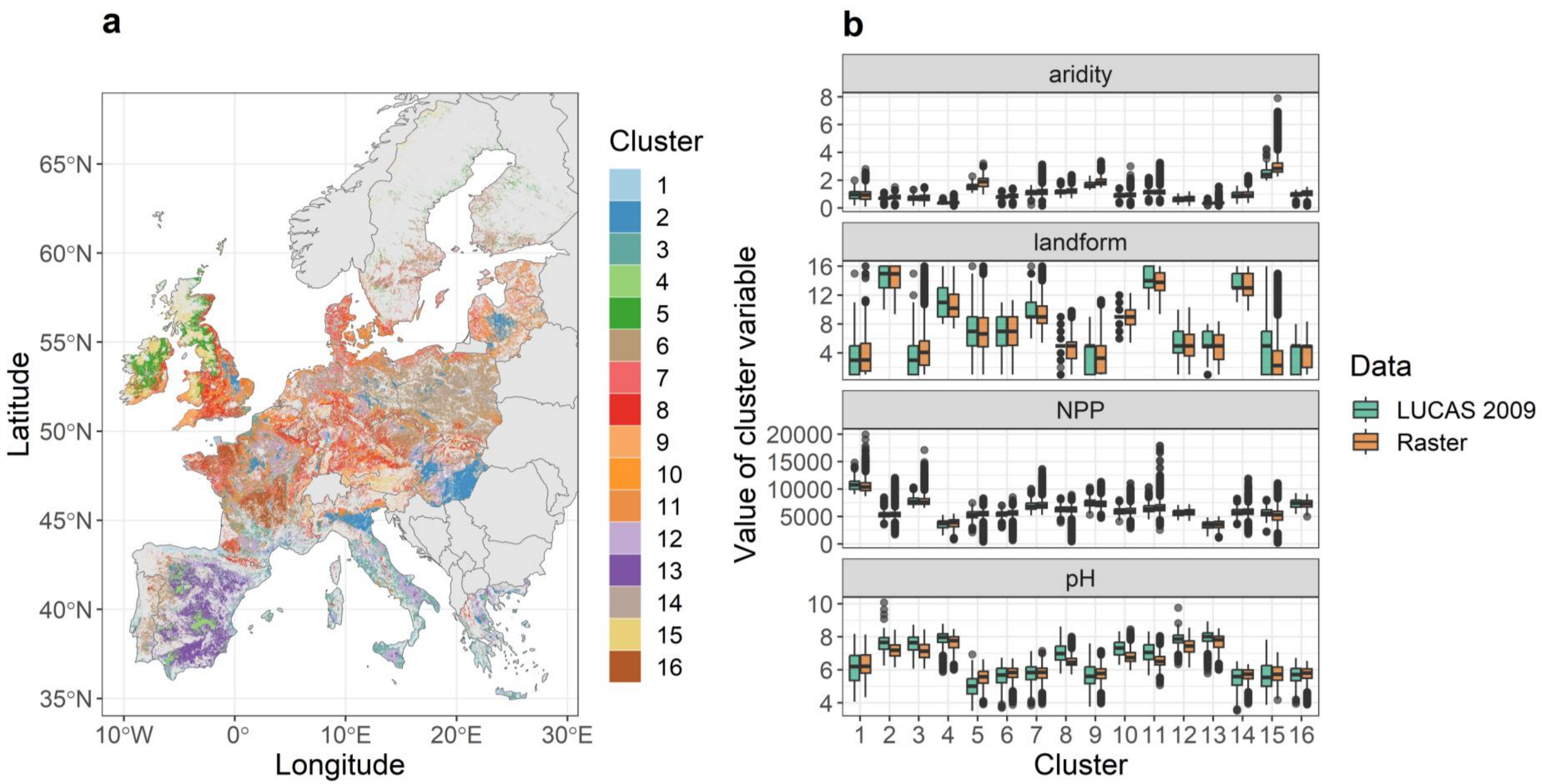


**Supplementary Figure 17 | a**. Random forest predicted cluster-associations for the raster that matches the extent of the ΔSOC raster[6] **b**. boxplots of the variables used for k-means (aridity, landform, net primary productivity (NPP) and soil pH by cluster associations plotted in their original scale. Boxplot colors indicate the LUCAS 2009 dataset (green) and the predicted raster data (orange). The center line of the boxplots is the median, the boxplot lower- and upper limits are equal to the first and third quartiles, respectively. The upper whisker extends 1.5 times the inter-quartile range from the upper limit, vice versa for the lower whisker. Vector map data used from the ‘rnaturalearth’ R package[20]. Copyright (CC0) (2025), (CRAN).

# Agreement in the SOC risk index class between regression methods

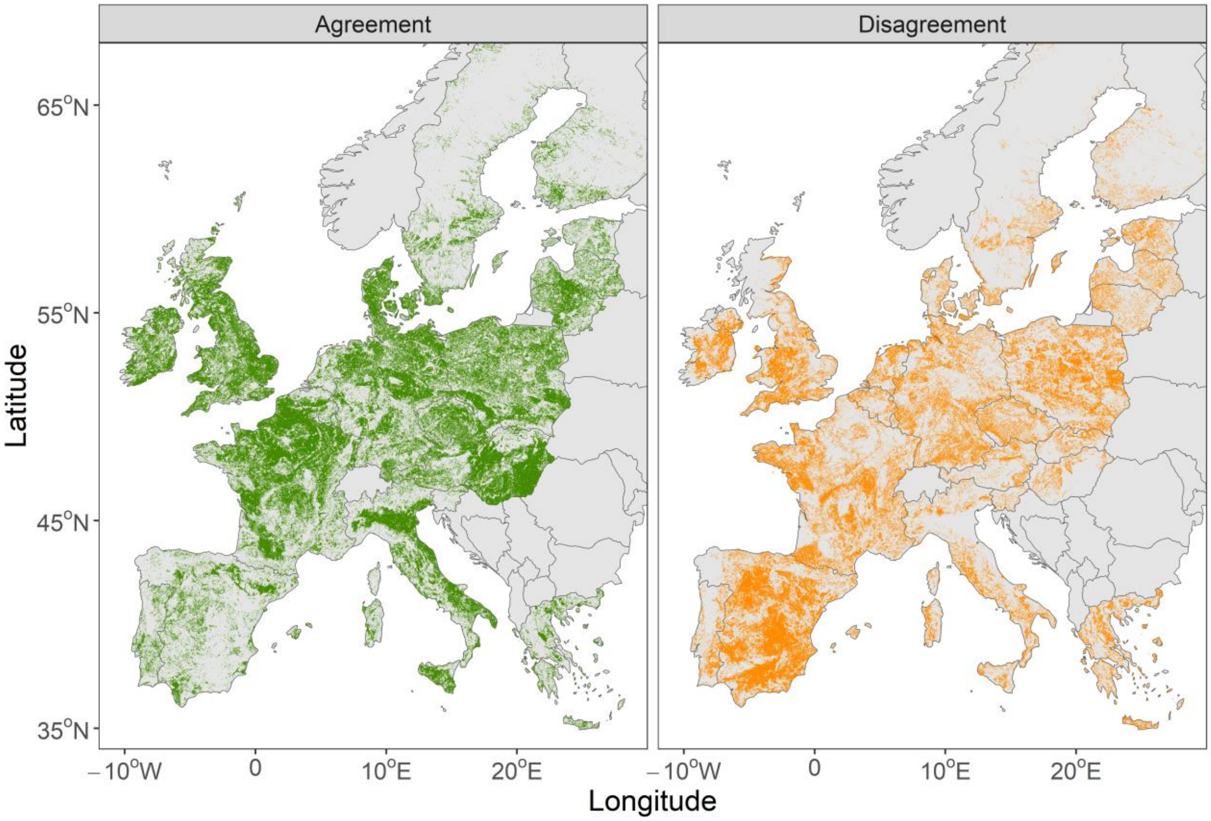


**Supplementary Figure 18 | Agreement in the soil organic carbon (SOC) risk index between methods to estimate effective mineral-associated organic carbon (MAOC) capacity.** This figure presents the locations where the three methods allocate a raster cell to the same SOC risk index class (see Fig. 5**b** and the methods section in main text). The regression methods are BL, PBL, and NBL (see Figure 3 in main text). Vector map data used from the 'rnaturalearth' R package[20]. Copyright (CC0) (2025), (CRAN).

## C losses and MAOC saturation (Georgiou et al. 2022)

The magnitude of saturation also determines the rate of soil C sequestration[19,21]. The SOC risk index presented in the main text is built on the concept that soils high in MAOC saturation lead to more rapid losses, as shown in a global synthesis[19]. This phenomenon is ascribed to higher POC content and weaker MAOC sorption/bonds to the mineral matrix[21–23]. Our data showed that high MAOC saturation also led to higher C losses on a continental scale, and was consistent for all three boundary line regression methods (Supplementary Figure 19 and Supplementary Table 3).

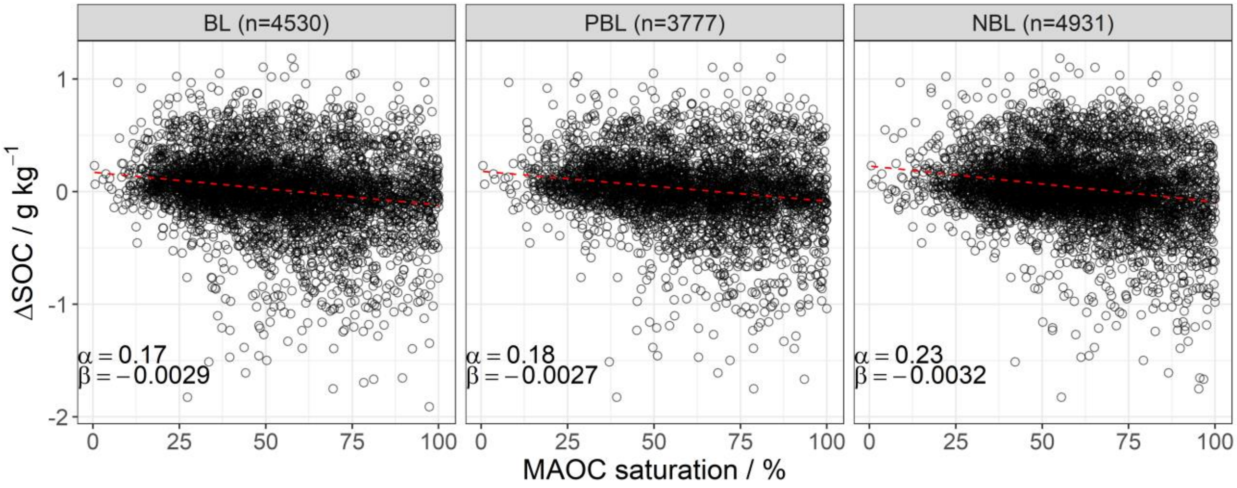


**Supplementary Figure 19 | Linear least squares model for regressing soil organic carbon changes (ΔSOC) against mineral-associated organic carbon (MAOC) saturation for each method to estimate the effective MAOC capacity (BL, PBL, NBL, see Fig. 3 in main manuscript).** To mitigate the effect of a truncated distribution at 100% MAOC saturation as a result of different fitting characteristics for coarse-textured soils (Figure 4 in main text), only data where MAOC saturation < 100% were included for creating this figure (see *n* for sample sizes). For a summary of parameter values, see Supplementary Table 3.

**Supplementary Table 3 | Summary table for linear model slope terms (α and β) accompanying Supplementary Figure 19** For details on the regression methods, see Figure 3 in the main text. SE the standard error, T-statistic the value to test whether the regression term is non-zero. P-value the two-side p-value associated with the observed t-statistic.

| Method | Term | Estimate | Std. error | T-statistic | p.value |
|---|---|---|---|---|---|
| BL | A | 0.17 | 0.0132 | 13.1 | 2.78E-38 |
| (n = 4530) | B | -0.0029 | 0.0002 | -12.9 | 2.43E-37 |
| PBL | A | 0.18 | 0.0150 | 12.1 | 3.63E-33 |
| (n = 3777) | B | -0.0027 | 0.0002 | -11.2 | 8.25E-29 |
| NBL | A | 0.23 | 0.0141 | 16.2 | 2.35E-57 |
| (n = 4931) | B | -0.0032 | 0.0002 | -14.3 | 1.56E-45 |

# Differences in C accrual potential between regression methods

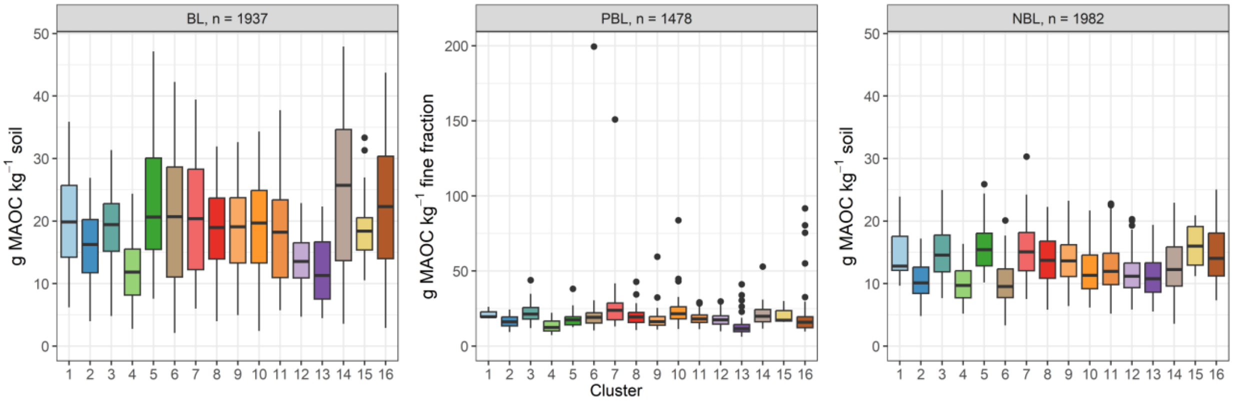


**Supplementary Figure 20 | Distance to the effective mineral-associated organic carbon (MAOC) capacity by each regression method and pedo-climatic cluster.** Distance to effective MAOC was calculated as: effective MAOC capacity – MAOC. For details on the regression methods, see Figure 3 in the main text. The data is the same subset as used for Supplementary Figures 5-7 although here it concerns only the samples categorized as no risk ('NR') and thus have the highest potential for soil C sequestration based on the proposed SOC risk index (n). Note that the PBL y-axis has a different range due to the different unit used in the PBL method. The center line of the boxplots is the median, the boxplot lower- and upper limits are equal to the first and third quartiles, respectively. The upper whisker extends 1.5 times the inter-quartile range from the upper limit, vice versa for the lower whisker.

## Local partial least squares regression method

The local partial least squares regression selects samples to include in the regression based on a similarity metric. We used the moving-window correlation as a metric to select *k*-nearest neighbors based on spectral similarity[24]. In order to choose the window size, we computed the RMSE between nearest neighbors for different window sizes (11-151 in steps of 10). We selected the window size with the lowest RMSE. For more details, see Ramirez-Lopez et al. (2013) and Summerauer et al. (2021)[9,24].

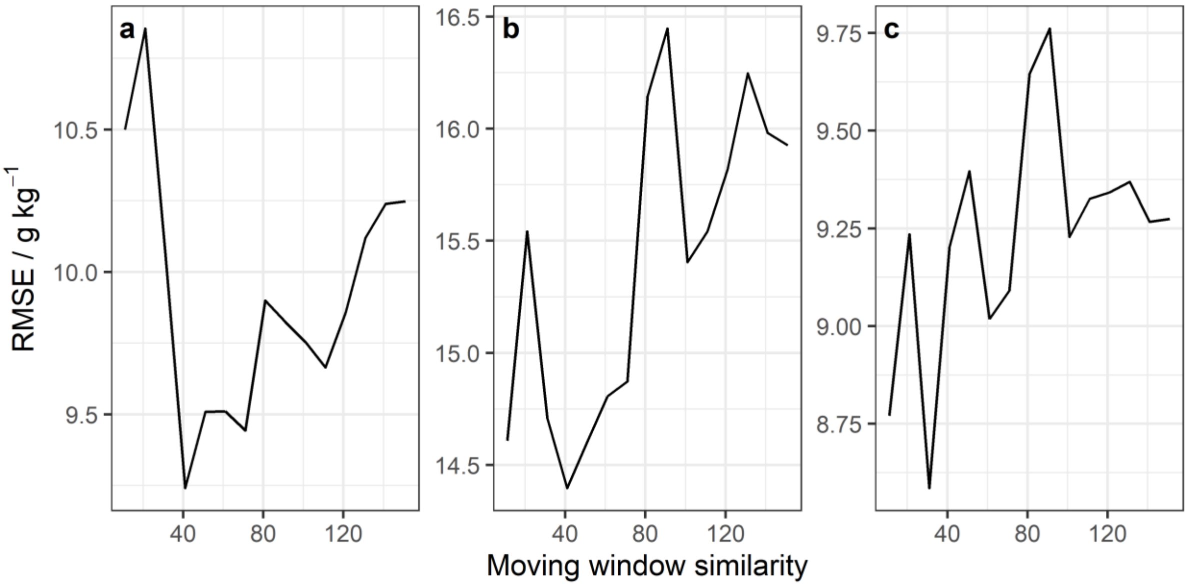


**Supplementary Figure 21 | The root mean squared error (RMSE) between nearest-neighbors by moving window size** for mineral-associated carbon (**a**), soil organic carbon (**b**), particulate organic carbon (**c**).

## Uncertainty propagation

We have performed an uncertainty propagation analysis based on the associated error with the MAOC predictions from the soil VNIR spectra. To assess the effect of marginal uncertainties in our MAOC predictions, we have approximated the expected error based on the predicted POC+MAOC vs. measured SOC (Extended Data Fig. 3). Given the negatively skewed distribution of SOC, we calculated the mean absolute log error (MALE). The MALE is robust to outliers (high SOC values). MALE reduces the effect of large differences between the predicted and measured values and provides a better measure of the relative difference. That is, the exponential of the MALE (EMALE) represents the relative multiplicative error (once 1 is subtracted). We assumed the error to be normally distributed around the mean prediction and that POC and MAOC contribute equally, so we divided the EMALE by two. We then performed 500 simulations where we resampled the mean MAOC prediction with a standard deviation (σ) represented by (EMALE-1) x MAOC. For each of these 500 realisations of MAOC, we then calculated the MAOC saturation and SOC index.

See Supplementary Figure 22a for the distribution of the standard deviations across the MAOC range. Supplementary Fig. 22b shows a histogram of the simulated distribution of MAOC values for a case where the mean MAOC is equal to 15.6 g $kg^{-1}$. The kernel density plots of the 500 MAOC simulations showed how for each simulation, the overall MAOC distribution approximated the density distribution of the MAOC as presented in the manuscript (Supplementary Fig. 22c).

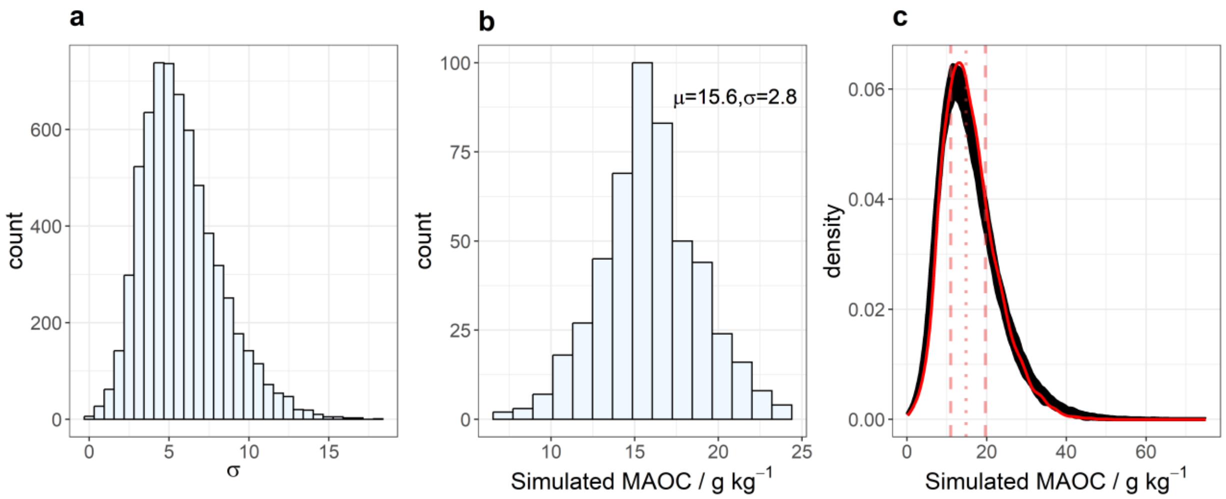


**Supplementary Figure 22 | Overview of the simulated mineral-associated organic carbon (MAOC) distributions based on the uncertainty propagation analysis.** Distribution of the standard deviation used in the simulation of prediction errors (**a**). Histogram of MAOC simulations for a single MAOC value, with the mean (μ) and standard deviation (σ) provided in text (**b**). Density distributions of the MAOC simulations (black lines) and the original distribution (red line). Dashed lines indicate the 1$^{st}$ quartile, median and 3$^{rd}$ quartile, respectively of the original distribution (**c**).

Based on the uncertainty propagation results, we calculated the 5$^{th}$ and 95$^{th}$ quantile for the MAOC saturation (Supplementary Fig. 23), and for the total areas corresponding to each SOC index class (Table 2 in main text).

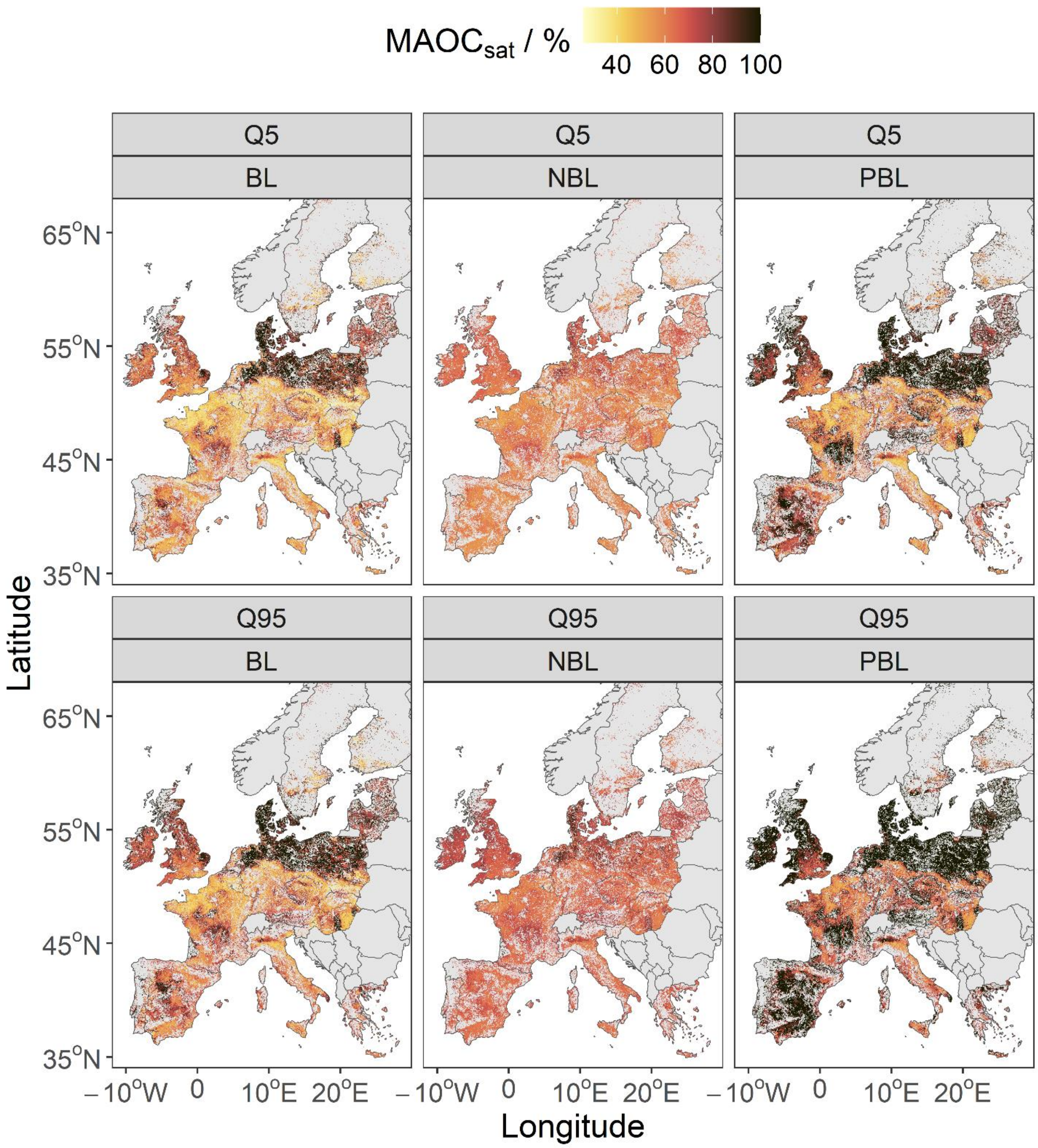


**Supplementary Figure 23 | Geographical representation of mineral-associated organic carbon (MAOC) degree of saturation (MAOC$_{sat}$).** Degree of MAOC saturation mapped to grid cells by cluster-specific MAOC saturation relationship for fine fraction bins. Values below 100% indicate a saturation deficit relative to the cluster-dependent effective MAOC capacity. Panels correspond to different methods to estimate the effective MAOC capacity (see Figure 3 in main text) and the 5$^{th}$ and 95$^{th}$ quantiles based on the uncertainty propagation analysis. The mean values are presented in Figure 5a in the main text. Vector map data used from the 'rnaturalearth' R package[20]. Copyright (CC0) (2025), (CRAN).

## ISO protocols for LUCAS 2009

**Supplementary Table 4 | ISO protocols used for analytical measurements in the LUCAS 2009 survey**, see references

| Soil property | ISO protocol |
|---|---|
| Clay, silt and sand / % | ISO 11277:1998[25] |
| pH in H2O / unitless | ISO 10390:1994[26] |
| Organic carbon / g $kg^{-1}$ | ISO 10694:1995[27] |

## Supplementary references

1. Toth, G, Jones, A, Montanarella, L, Alewell, C. LUCAS Topoil Survey-methodology, data and results. (2013).

2. Lugato, E., Lavallee, J. M., Haddix, M. L., Panagos, P. & Cotrufo, M. F. Different climate sensitivity of particulate and mineral-associated soil organic matter. *Nat. Geosci.* **14**, 295–300 (2021).

3. Abatzoglou, J. T., Dobrowski, S. Z., Parks, S. A. & Hegewisch, K. C. TerraClimate, a high-resolution global dataset of monthly climate and climatic water balance from 1958-2015. *Sci. Data* **5**, (2018).

4. Iwahashi, J. & Pike, R. J. Automated classifications of topography from DEMs by an unsupervised nested-means algorithm and a three-part geometric signature. *Geomorphology* **86**, 409–440 (2007).

5. Running, S., Zhao, M. MODIS/Terra Net Primary Production Gap-Filled Yearly L4 Global 500m SIN Grid V061. 2021. *NASA EOSDIS Land Processes DAAC* https://doi.org/10.5067/MODIS/MOD17A3HGF.061 (2021).

6. De Rosa, D. *et al.* Soil organic carbon stocks in European croplands and grasslands: How much have we lost in the past decade? *Glob. Chang. Biol.* **30**, (2024).

7. Kennard, R. W. & Stone, L. A. Computer Aided Design of Experiments. *Technometrics* **11**, 137–148 (1969).

8. Minasny, B. & McBratney, A. B. Regression rules as a tool for predicting soil properties from infrared reflectance spectroscopy. *Chemom. Intell. Lab. Syst.* **94**, 72–79 (2008).

9. Ramirez-Lopez, L. *et al.* Distance and similarity-search metrics for use with soil vis-NIR spectra. *Geoderma* **199**, 43–53 (2013).

10. Lin, L. I.-K. A Concordance Correlation Coefficient to Evaluate Reproducibility. *Biometrics* **45**, 255 (1989).

11. Bellon-Maurel, V., Fernandez-Ahumada, E., Palagos, B., Roger, J. M. & McBratney, A. Critical review of chemometric indicators commonly used for assessing the quality of the prediction of soil attributes by NIR spectroscopy. *TrAC - Trends in Analytical Chemistry* vol. 29 1073–1081 at https://doi.org/10.1016/j.trac.2010.05.006 (2010).

12. Martens, H. & Næs, T. Multivariate Calibration. in *Chemometrics* 147–156 (Springer Netherlands, 1984). doi:10.1007/978-94-017-1026-8_5.

13. Leifeld, J. Application of diffuse reflectance FT-IR spectroscopy and partial least-squares regression to predict NMR properties of soil organic matter. *Eur. J. Soil Sci.* **57**, 846–857 (2006).

14. Dangal, S. R. S., Sanderman, J., Wills, S. & Ramirez-Lopez, L. Accurate and precise prediction of soil properties from a large mid-infrared spectral library. *Soil Syst.* **3**, 1–23 (2019).

15. Viscarra Rossel, R. A. *et al.* How much organic carbon could the soil store? The carbon sequestration potential of Australian soil. *Glob. Chang. Biol.* **30**, (2024).

16. Feng, W., Plante, A. F. & Six, J. Improving estimates of maximal organic carbon stabilization by fine soil particles. *Biogeochemistry* **112**, 81–93 (2013).

17. Six, J., Doetterl, S., Laub, M., Müller, C. R. & Van De Broek, M. The six rights of how and when to test for soil C saturation. *SOIL* **10**, 275–279 (2024).

18. Begill, N., Don, A. & Poeplau, C. No detectable upper limit of mineral-associated organic carbon in temperate agricultural soils. *Glob. Chang. Biol.* **29**, 4662–4669 (2023).

19. Georgiou, K. *et al.* Global stocks and capacity of mineral-associated soil organic carbon. *Nat. Commun.* **13**, (2022).

20. Massicotte, P. & South, A. rnaturalearth: World Map Data from Natural Earth. *R package version 0.3.2* https://cran.r-project.org/package=rnaturalearth (2023).

21. West, TO, Six, J. Considering the influence of sequestration duration and carbon saturation on estimates of soil carbon capacity. *Clim. Change* **80**, 25–41 (2007).

22. Gulde, S., Chung, H., Amelung, W., Chang, C. & Six, J. Soil Carbon Saturation Controls Labile and Stable Carbon Pool Dynamics. *Soil Sci. Soc. Am. J.* **72**, 605–612 (2008).

23. Kleber, M. *et al.* Mineral-Organic Associations: Formation, Properties, and Relevance in Soil Environments. *Adv. Agron.* **130**, 1–140 (2015).

24. Summerauer, L. *et al.* The central African soil spectral library: A new soil

infrared repository and a geographical prediction analysis. *SOIL* **7**, 693–715 (2021).

25. ISO. 11277. https://www.iso.org/standard/19255.html (1998).

26. ISO. 10390. https://www.iso.org/standard/18454.html (1994).

27. ISO. 10694. https://www.iso.org/standard/18782.html (1995).
